# Effectiveness of low-level laser therapy in reducing pain score and healing time of recurrent aphthous stomatitis: a systematic review and meta-analysis

**DOI:** 10.1186/s13643-024-02595-0

**Published:** 2024-07-22

**Authors:** Desiana Radithia, Fatma Yasmin Mahdani, Reiska Kumala Bakti, Adiastuti Endah Parmadiati, Ajiravudh Subarnbhesaj, Selviana Rizky Pramitha, I Gusti Agung Sri Pradnyani

**Affiliations:** 1 Department of Oral Medicine, Faculty of Dental Medicine, Universitas Airlangga, Jl. Mayjen Prof. Dr. Moestopo No.47, Pacar Kembang, Kec. Tambaksari, Kota SBY, Jawa Timur 60132 Indonesia; 2 Department of Oral Biomedical Science, Division of Oral Diagnosis, Faculty of Dentistry, Khon Kaen University, 123 Thanon Mittraphap, Tambon Nai Mueang, Mueang Khon Kaen District, Khon Kaen, 40002 Thailand; 3 Oral Medicine Specialist Study Program, Faculty of Dental Medicine, Universitas Airlangga, Jl. Mayjen Prof. Dr. Moestopo No.47, Pacar Kembang, Kec. Tambaksari, Kota SBY, Jawa Timur 60132 Indonesia

**Keywords:** Healing time, Low-level laser therapy, Pain score, Recurrent aphthous stomatitis

## Abstract

**Background:**

Recurrent aphthous stomatitis (RAS) is a common chronic inflammatory oral disease that negatively impacts the quality of life. Current therapies aim to reduce pain and healing process yet challenges such as rapid loss due to salivary flushing in topical drugs and adverse effects due to prolonged use of systemic medications require further notice. Low-level laser therapy is reported with immediate pain relief and faster healing thus preserving the potential for optimal treatment modalities. This review critically analyses and summarizes the effectiveness of LLLT in reducing pain scores and healing time of RAS.

**Methods:**

A systematic search was conducted in ScienceDirect, PubMed, and Scopus using keywords of low-level laser therapy, photo-biomodulation therapy, and recurrent aphthous stomatitis. RCTs between 1967 to June 2022, presenting characteristics of the laser and reporting pain score and/or healing time of RAS after irradiation were included. Animal studies and recurrent aphthous ulcers with a history of systemic conditions were excluded. Studies were critically appraised using the RoB 2 tool. A meta-analysis was performed using inverse variance random effects.

**Results:**

Fourteen trials with a total of 664 patients were included. Reduced pain was reported in 13 studies, while shortened healing time was presented in 4. The pooling of two studies after CO2 irradiation demonstrated faster healing time compared to placebo (MD − 3.72; 95% CI − 4.18, − 3.25).

**Conclusion:**

Pain score and healing time of RAS were reduced after irradiation with LLLT. RoB resulted in “some concerns” urging well-designed RCTs with larger samples to further assess each laser application for comparison.

**Systematic review registration:**

PROSPERO CRD42022355737.

**Supplementary Information:**

The online version contains supplementary material available at 10.1186/s13643-024-02595-0.

## Background

Recurrent aphthous stomatitis (RAS), also known as canker sores, is a common inflammatory oral condition that presents as painful round to oval-shaped ulcers with a well-defined border surrounded by erythematous area and covered by pseudomembranous base affecting non-keratinized oral mucosa [1, 2]. This condition occurs in 20% of the global population ranging from 5 to 60% based on study and population. In Indonesia, national prevalence reported the prevalence of RAS at 8 to 12% including 45.42% among the South Kalimantan population, 48% among prisoners, and 68% among dental students [3–5]. The major symptom of RAS is pain that impairs nutritional and water intake, possibly leading to subsequent debilitating dehydration. Annual reoccurrence can be seven or more episodes, equating to 27% of a given year that a patient may experience discomfort associated with the disease. This emphasizes the need to manage the ulcers before compromising the well-being of a person [6, 7].

Three principles in the treatment of RAS are to decrease symptoms, reduce ulcer size and number, and increase the ulcer-free period [8]. Glucocorticoids and antimicrobial therapy are considered the conventional treatment for RAS. These medications have been applied as topical pastes, mouth rinses, and intralesional injections. However, there are currently many therapeutic challenges, including low drug efficacy and poor retention at the targeted site of action [9]. Severe and constantly recurring ulcerations were indications for systemic therapy. However, such approaches can generate severe side effects ranging from somnolence to nausea and gastrointestinal symptoms [10]. Several innovative drug delivery systems have been developed for the local treatment and the prevention of various diseases in the oral cavity, yet the efficacy in reducing the size and the number of ulcers was only achieved in 50 to 62% of cases [9]. No improvement after 14 days of treatment was also reported, which urges the need for another remedy unaffected by salivary wash-out and presented systemic side effects with higher efficacy should be evaluated [10, 11].

Low-level laser therapy (LLLT) or cold laser is non-destructive energy that occurs at the periphery of the target tissue with a wavelength from 630 to 1100 nm and a power between 2 and 200 mW [12, 13]. It has bio-stimulating effects, thereby accelerating tissue healing, presenting anti-inflammatory effects on target cells and tissues, and reducing pain from various aetiologies [12, 14]. Several different types of laser (Nd:YAG laser, CO2 laser, and diode laser, etc.) have been applied as the treatment of RAS and they indeed demonstrated superiority in pain relief and faster healing compared to placebo or medical treatment group [14]. The benefit of LLLT includes its short-term local application in contact or non-contact mode thus unaffected by salivary wash-out and not presenting with any systemic side effect [15–17].

Previous reviews summarized the effect of LLLT in treating RAS using a systematic approach, nonetheless, a study to analyze the efficacy and estimate the effect of LLLT compared to other therapies is essential for consideration in utilizing this treatment in clinical settings [18–22]. Based on preliminary searches in databases (PubMed, ScienceDirect, and Scopus) and a registry for systematic review protocol (PROSPERO), this is the first review to study the effects of intervention and the first meta-analysis for low-level laser therapy to assess the reduction of pain and healing time in RAS.

## Method

This review follows the Preferred Reporting Items for Systematic Review and Meta-Analysis (PRISMA) 2020, the 6.3 version of the Cochrane Handbook for Systematic Review of Interventions [20, 23, 24]. Prospective registration of the review protocol was submitted to PROSPERO with registration number CRD42022355737 to help minimize bias in the conduct and reporting of the review, reduce duplication of effort between groups, and keep the previous systematic reviews updated [25].

### Eligibility criteria

The inclusion and exclusion criteria of the studies in this review are detailed using the PICOS (Population; Intervention; Comparator; Outcome(s); Study design) framework [20].

#### Types of population

This review included studies that examine the effect of low-level laser therapy as the treatment of recurrent aphthous stomatitis in patients of any age. Recurrent aphthous stomatitis is defined as an inflammatory condition of oral mucosal surface in the form of painful small round ulcers that reappear from the mouth from time to time in non-movable/non-keratinizing mucosa without a history of other systemic diseases [26, 27]. A trained individual evaluated participants with recurrent aphthous stomatitis from full history taking and clinical examination based on the 1972 Stanley Classification of Recurrent Aphthous Stomatitis [28, 29]. There are three clinical presentations of RAS: Minor RAS, major RAS, and herpetiform ulceration. Minor RAS is superficial, usually < 1 cm in diameter, and their size is approximately 4–5 mm in diameter. Major RAS are similar in appearance to those of minor RAS; however, they are larger than 10 mm in diameter, are deeper, often scarred, and can last for weeks to months. Herpetiform ulcers are small (1–2 mm), and multiple ulcers (5–100) may be present at the same time. Animal studies and recurrent aphthous ulcers with a history of systemic conditions were excluded.

#### Types of intervention

Low-level laser therapy uses coherent light sources (lasers) or non-coherent light sources consisting of filtered lamps, light emitting diodes (LED) or a combination of both with power ranges from 1 to 500 mW with a wavelength range from 300 to 10,600 nm. Laser parameters are mainly reported effective at a wavelength from 630 to 1100 nm and with a power between 2 and 200 mW [12, 13, 30, 31].

#### Types of comparators

The comparator in this review included other therapies, namely placebo and conventional therapy. Placebo included passive laser, which was applied at the same procedure in the treatment group but without any power about which the patient was not aware or with no intervention. Meanwhile, conventional therapy included topical or systemic application of pharmacotherapy such as antimicrobial agents, anesthesia, anti-inflammatory drugs, immunomodulators, or corticosteroids [8, 11].

#### Types of outcomes measured

Articles presenting pain intensity (reported in pain scale or score) immediately and 1 day after therapy as well as the healing time of the ulcer (reported in days) were included in this review if present.

#### Types of study

Randomized controlled trials (RCTs) were included, comprising quasi-RCTs (when the allocation of participants may be based on alternation, date of birth, or case record number) and RCTs with an open-label study design where investigators and patients are aware of the intervention given. No language restriction was imposed in this review.

### Search methods for identification of studies

In the search strategy, a combination of Medical Subject Headings (MeSH) terms and keywords was applied: (low-level laser therapy OR phototherapy laser OR biostimulation laser OR photobiomodulation therapy) AND recurrent aphthous stomatitis. A validated RCT filter was used to exclude studies with different designs. A literature search was performed in the following electronic databases from their inception date from 1 January 1967 to 30 June 2022: ScienceDirect, PubMed, and Scopus.

**Fig. 1:**
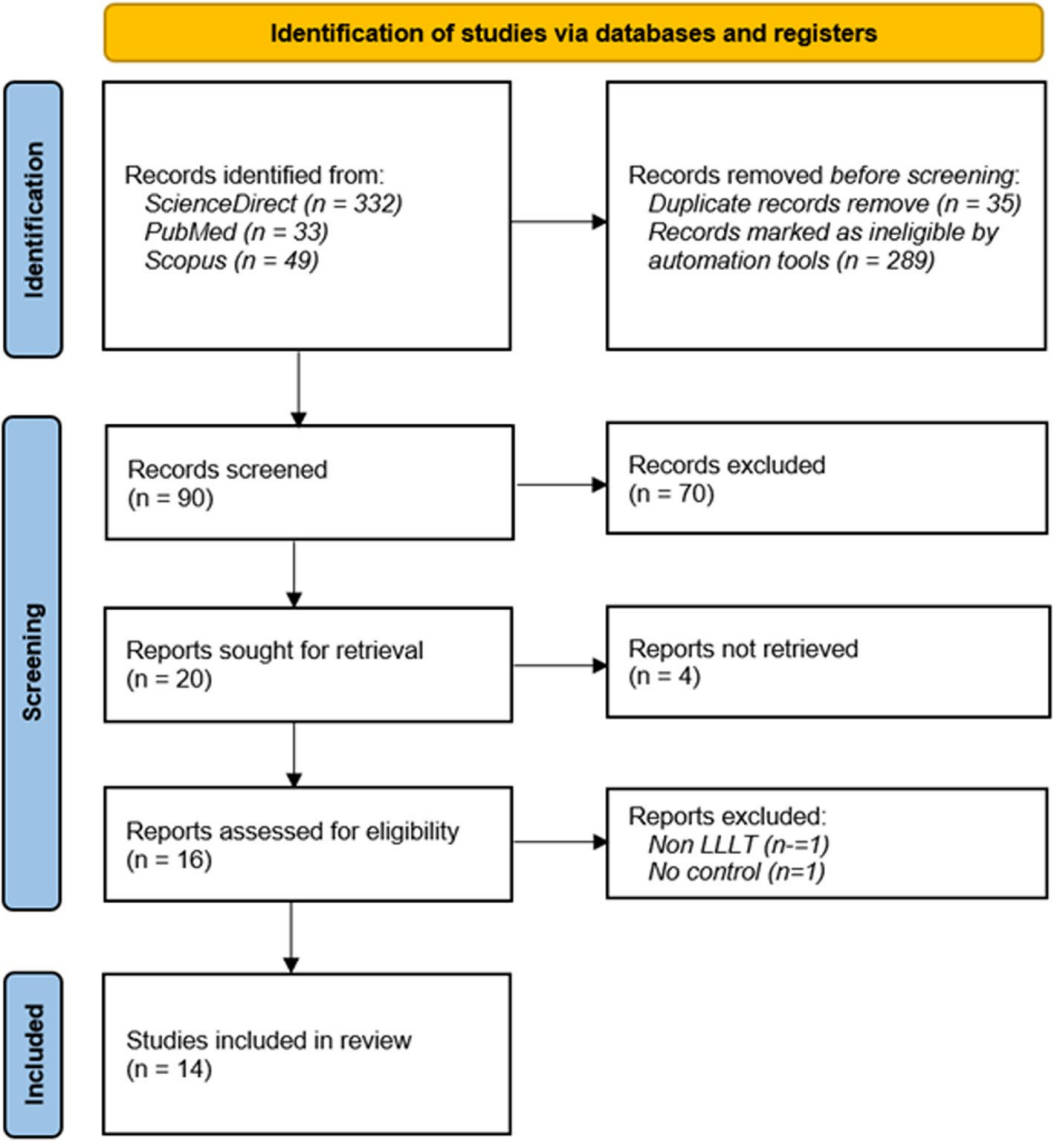
Flow diagram based on PRISMA 2020

Figure 1 illustrates the search and selection process, which includes identification, inclusion, and process of exclusion. In the identification stage, automation tools removed articles after screening for duplication and ineligibility. Records to be screened were excluded based on title relevancy with inclusion criteria. After the exclusion of the irrelevant title, reports were assessed for relevant information in the abstract. Following the exclusion based on irrelevant abstract, records were further assessed for the eligibility criteria.

### Data collection and analysis

Titles and abstract searches were independently performed by three reviewers (SRP/FYM/IGASP) and assessed to obtain relevant full-text articles for the review. Data were further extracted from the included articles using the predefined form, which was previously prepared in a spreadsheet by SRP and FYM after consulting with DR and AEP. Data extraction form includes author, year, characteristics of participants, characteristics of intervention (including low-level laser therapy [type of laser, wavelength, mode, output power, exposure time, total energy, application of gel, application distance] and other therapies namely topical medication [types of medication, times of application], and placebo), outcome measures, and statistical result. All data were double-checked by SLV for accuracy after the initial abstraction. Any disagreements were resolved between reviewers. When consensus was not achieved, reviewers consulted disagreements with DR as appropriate. Additional information was obtained from the original RCTs, other online supplementary material, or by contacting study authors (Supplementary material 1 and 2).

### Assessment of methodological quality of included reviews

Risk of bias was independently assessed by three reviewers (SRP/FYM/RKB) for each included trial using the Cochrane 'Risk of bias' 2 tool. We resolved disagreements by discussion between the reviewers. When consensus was not achieved, reviewers consulted disagreements with DR [20]. The overall quality of the evidence for each outcome was evaluated independently using the GRADE approach. The GRADE approach improves reliability in comparison to intuitive judgments about the certainty of a body of evidence [32].

### Data synthesis

This review deployed narrative synthesis for pain score as the outcome measures. The diversity of scale used for outcome measures and the absence of standard deviation in study results prevented the input from meta-analysis for pain score.

Studies presenting healing time were assessed using the standard meta-analysis (direct comparisons) performed using Review Manager 5.4 to determine the effectiveness of treatments directly compared to each other. The *I*^2^ statistic was used to quantify the heterogeneity of the results in individual studies since heterogeneity is a common issue encountered while performing meta-analyses. The random-effects model was applied as the default option for illustrative purposes in all the forest plots presenting effect measure data per treatment. MD or mean difference was used for continuous outcomes such as healing time. Data analysis was performed by SLV.

Subgroup analyses were considered in reporting the continuous effect of healing time by splitting intervention based on the type of laser wavelength. Sensitivity analysis by excluding studies with a high risk of bias could not be performed as the number of studies is limited while mixing all types of lasers into one estimated effect and splitting based on the laser dosage may help detect changes in the findings [20].

## Result

### Description of study

#### Results of the search

A total of 414 papers were identified. Ninety records were screened after removing duplicates (35) and ineligible reports marked by an advance filter for RCTs (289). Seventy-four articles were excluded after title and abstract evaluation, and sixteen reports were retrieved for eligibility assessment. The full-text assessment resulted in two papers discarded and 14 RCTs to be reviewed.

### Included studies

#### Study population

Fourteen studies involving 664 participants, ranging from 15 to 180 [33] participants [33, 34], were included. Only one study enrolled more than 100 patients [33]. Participants included in this review ranged from 1 year old to 70 years old with three studies not reporting the age of participants. Gender distribution demonstrated males with a total of 156 participants and females with a total of 320 participants. Three studies did not report the characteristics of the participants based on age [12, 14, 35] and four studies did not report the characteristics of the participants based on gender [12, 36–38].

Two studies used the history of aphthous ulcer and clinical manifestation in diagnosing RAS followed by laboratory testing and pathergy test to eliminate the presence of any systemic disease [39, 40]. One study reported using history taking, clinical manifestation, and laboratory testing only [36]. Ten studies reported the diagnosis of RAS based on the history of disease and clinical manifestation, yet not mentioning which classification or characteristics of ulcer were used [14, 34, 35, 37, 38, 41–44]. Only one study mentioned the use of the 1972 Stanley Classification of RAS, while the other two did not mention the method to diagnose RAS [12, 33]. Twelve studies reported the use of intervention and control in minor RAS, while the other two did not mention the type of RAS.

#### Study design and setting

All trials were randomized where five trials were single-blinded [35–37, 39, 41], three trials were double-blinded [38, 40, 44] and six trials not reporting the blinding of participants and evaluator [12, 14, 33, 34, 42, 43]. Based on the study setting, four studies were conducted in Iran [37–40], and the others were each from Brazil [34], India [36], Sweden [41], Bulgaria [33], Turkey [35], Malaysia [42], Egypt [43], Italy [44], China [14], and Iraq [12]. Summary for study population and study design presented is presented in Table 1.

**Table 1:**
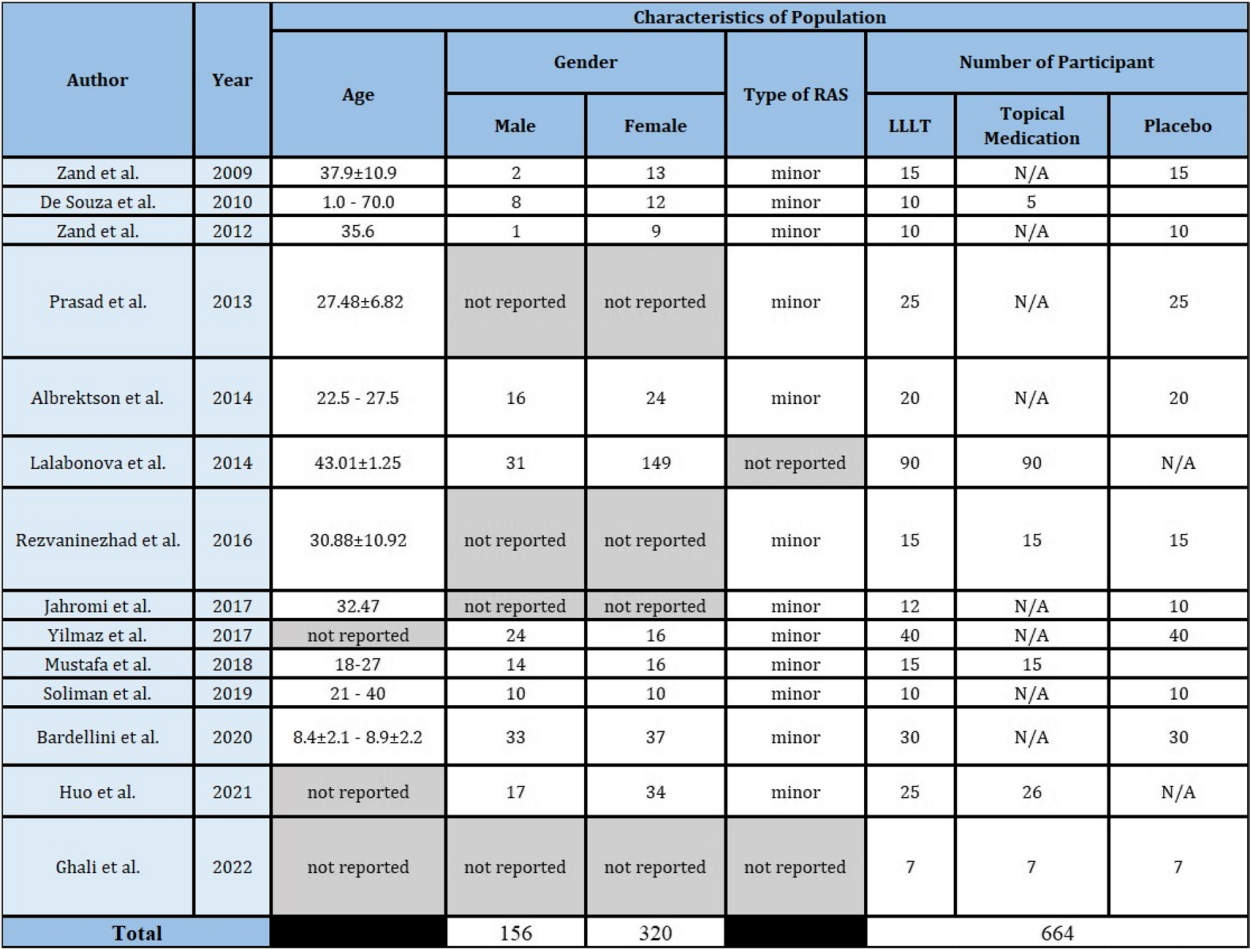
Characteristics of population and study design

*N/A* not applicable

#### Intervention and comparators

Four studies reported CO2 laser [36, 37, 39, 40], while the other four used diode laser [12, 14, 43, 44] as an intervention. Two studies reported the use of InGaAIP [34, 38], two studies used Er,Cy: YSGG [35, 42], one study used GaAIAs [41], and one study used Nd: YAG [33]. Eight studies were placebo-controlled (sham treatment) [12, 35–41, 43, 44], four were in comparison with topical medication (three with glucocorticoid and one with wound healing promoting agent) [14, 33, 34, 42], and two studies were in comparison with both topical medication and placebo (one with glucocorticoid and sham treatment [37] while the other one with antimicrobial and motivation only [12]). Summary for studies intervention and comparators is presented in Table 2.

**Table 2:**
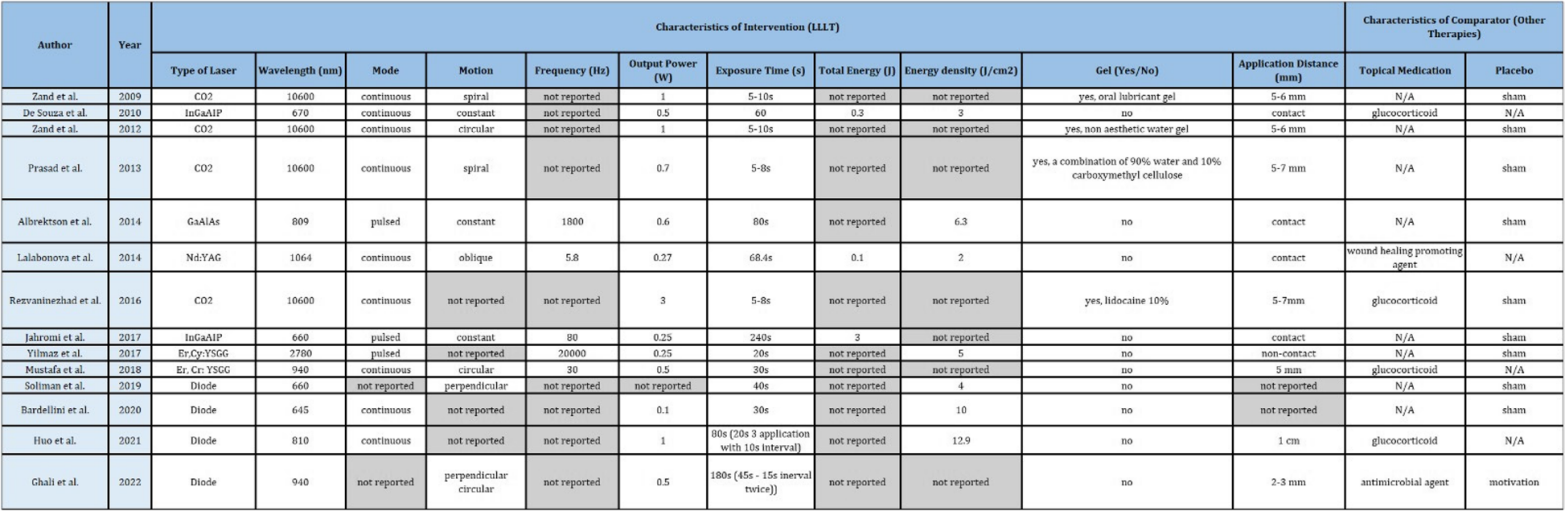
Characteristics of intervention and comparators

*N/A* not applicable

#### Outcomes

Outcomes varied in terms of measurement and reporting. Pain scores were reported on the scale (VAS 10 in eight studies [14, 33, 35, 38, 39, 43, 44], VAS 100 in three studies [12, 37, 41], NRS 11 in two studies [36, 42], and Pain Intensity Score in one study [34]). Six studies presented the result as mean value with standard deviation (one study presented the description only) [14, 35, 37, 39, 43, 44], three studies presented mean value only [12, 36, 41], one study presented mean difference [42], one study presented percentage of pain-free patient [38], one study presented in percentage of pain reduction [33], and one study not providing pain intensity outcome [40].

Healing time was also reported in various parameter measurements in which three studies reported in days [14, 36, 40] and one study in healing of RAS (HRAS) score [35]. For meta-analysis to estimate the effect of LLLT on healing time, we removed one study due to the absence of mean value and standard deviation in HRAS score.

### Excluded studies

Two studies were excluded for the following reasons: they were non-LLLT or presented an ablative and thermogenesis effect [45] and no control [46].

### Risk of bias in included studies

**Fig. 2:**
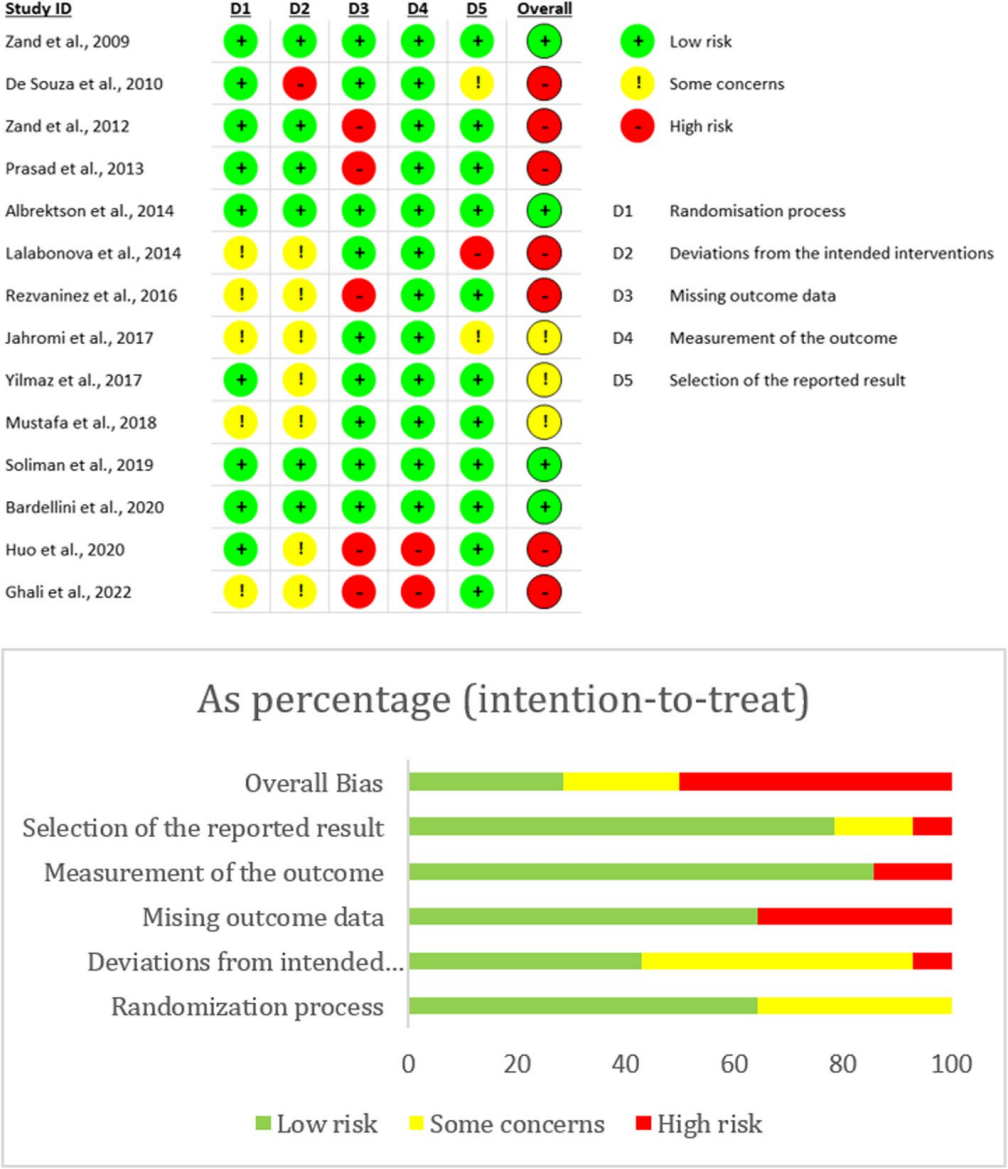
Risk of bias summary of each included study and risk of bias in percentage across all included studies

The risk of bias in all included studies was summarized in Fig. 2. Four studies scored low risk across all domains [39, 41, 43, 44], three studies scored some concerns [35, 38, 42], and seven studies scored high risk (five in one domain and two in multiple domains) [33, 34, 36, 37, 40]. However, the overall risk for all studies was considered to be in “some concerns” because susceptibility to bias resulted from no explicit report of the domain, and no differences were observed in baseline results between intervention and comparator groups [20].

### Effect of intervention

Based on Table 3, it can be seen that 13 studies described the reduction in pain score [12, 14, 33–39, 41–44]. Eight studies reported a significant reduction in pain scores within the group after applying LLLT [14, 34–36, 41–44], and five studies did not report statistical analysis within the group [12, 33, 37–39]. Statistical analysis within the group presented significant pain reduction immediately after LLLT application in two studies [36, 39] and 1 day after LLLT application in four studies [14, 42–44]. Significant pain reduction both immediately and 1 day after the application of LLLT was reported in two studies [35, 41].

**Table 3:**
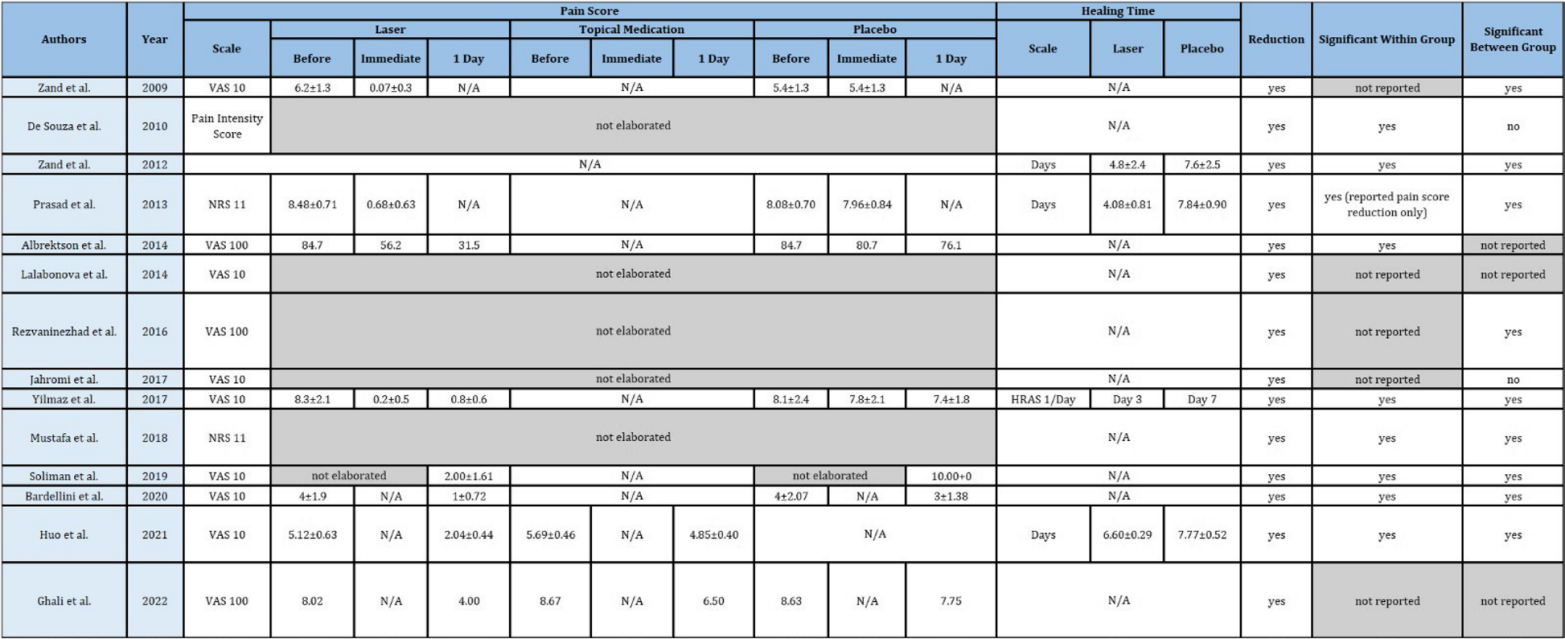
Characteristics of outcomes and “summary of findings”

*N/A* not applicable

Compared to topical medication, three studies reported a significant reduction in pain score [14, 37, 42], and two studies did not report statistical analysis for a significant reduction between groups [12, 33]. Compared to placebo, six studies reported significant pain reduction between laser and placebo groups [35–37, 39, 43, 44], while one study reported no significant reduction [38]. Two studies reported no statistical analysis between groups [12, 41].

Shortened healing time is reported in four studies where significant differences between the laser group and the control group were reported statistically [14, 35, 36, 40]. Three studies resulted in significant differences in healing time when compared to placebo [35, 36, 40]. One study reported a significant difference when compared to topical medication [14].

**Fig. 3:**
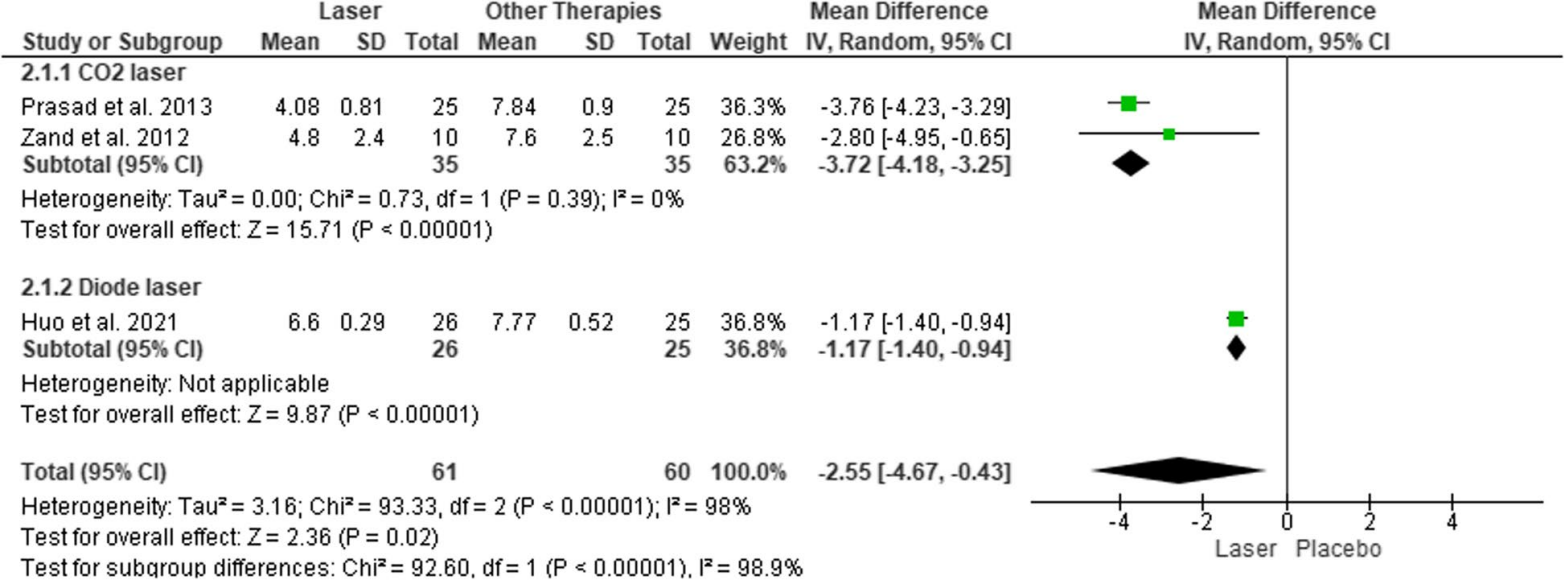
Forest plot for estimating the effect of LLLT on healing time

Meta-analysis was performed in three studies [35, 36, 40] with an inverse variance random effect because healing time is continuous data (changing over time), and a mean difference was obtained considering that all studies used the same parameter to measure healing time in days [20]. The overall analysis presented a reduction in healing time after the application of LLLT compared to topical medication and placebo (− 2.55 [CI 95% − 4.67, − 0.43]) with high heterogeneity (*I*^2^ = 98%). Subgroup analysis reported a reduction of healing time after application of CO_2_ laser compared to placebo (− 3.72 [CI 95% − 4.67, − 0.43]) with low heterogeneity (*I*^2^ = 0%). Subgroup analysis for diode laser cannot be performed due to a limited number of trials but a single MD also showed a reduction in healing time (− 1.17 [CI 95% − 1.40, − 0.94]).  Estimated effect of LLLT on healing time is presented in Fig. 3.

The overall quality of evidence for reduced healing time was assessed using GRADE, as presented in Table 4. The evidence was initially graded as “high” since all studies were randomized controlled trials. Due to crucial limitations for one or multiple risks of bias domains that are sufficient to lower confidence in the estimate of effect in comparison to topical medication and placebo, such as not explicitly reporting randomization process, deviations from the intended interventions, measurement of the outcome and selection of the reported result, the quality was downgraded two levels to “low”. Evidence of CO_2_ laser application in reducing healing time was downgraded one level to “moderate” because there is a crucial limitation in not explicitly reporting any missing outcome data.

**Table 4:** Summary of findings for the main comparison: healing time

**Comparisons**	**Number of participants (studies)**	**Quality of the evidence (GRADE)**	**Anticipated mean difference (95% CI)**
Low-level laser therapy (LLLT) vs conventional therapy (topical medication and placebo)	121 (3 studies)	⊕⊕◯◯ Low	− 2.55 [95% CI − 4.67, − 0.43]; *I*^2^ = 98%
CO2 vs placebo	70 (2 studies)	⊕⊕⊕◯ Moderate	− 3.72 [95% CI − 4.18, − 3.25]; *I*^2^ = 0%

*High quality* further research is very unlikely to change our confidence in the estimate of effect, *Moderate quality* further research is likely to have an important impact on our confidence in the estimate of effect and may change the estimate, *Low quality* further research is very likely to have an important impact on our confidence in the estimate of effect and is likely to change the estimate, *Very low* quality we are very uncertain about the estimate

## Discussion

Recurrent aphthous stomatitis (RAS) is a mucosal lesion resulting from a T cell-mediated immunologic reaction that may develop from an erythematous macule to round well-defined ulcers surrounded by an erythematous border [1, 2]. The mechanism of ulcer formation in RAS includes activating T helper cells after heat shock protein presentation by antigen-presenting cells in lymph nodes. This antigen will be cross-presented to T cytotoxic cells via IL-2 production from activated T helper cells and MHC class I presentation in APC after CD40-CD40 ligand binding. T cell cytotoxic will migrate to the affected site and cause mucosal destruction via the synthesis of perforin and granzyme. Endogenous mediators released from the damaged tissues increase the extravasation of the vessels and attract the immune cells, including mast cells, macrophages, neutrophils, and platelets, to the injured site for the inflammatory response [47, 48]. Inflammatory mediators produced from this response can directly activate the nociceptors, evoking pain or modulating the sensitivity of the primary nociceptors, thus causing a hyperreactive reaction to stimuli [49]. Focusing on the pathogenesis of RAS, pain becomes the primary symptom reported in this review using VAS scores ranging from 4 to 8 [12, 14, 33–39, 41–44]. This condition usually lasts 7 to 21 days before healing spontaneously and may recur up to more than seven episodes in a year, thus affecting individual well-being [6, 7, 26]. Treatments are frequently investigated to reduce pain, decrease the size of the lesion, and prevent reoccurrence [8]. Accelerated wound healing may also target tissue repair, preventing direct contact of external stimuli to nerve endings in lamina propria due to ulcer formation [50].

This review included 14 studies investigating the effect of low-level laser therapy on RAS, mainly minor RAS. Patients with minor RAS should be reassured about disease development; medication is only prescribed when required [51]. Reassurance or motivation is typically provided in the form of accurate or potentially corrective verbal information that intends to reduce the threat value of disease, such as thoughts and beliefs about pain, that can reduce pain intensity [52]. When there is a symptom of exacerbation, topical glucocorticoids (GCs) are the first-line drug for minor RAS. They are used for local treatment through their anti-inflammatory and immunosuppressive effects [9, 53, 54]. Glucocorticoids suppress pain intensity by directly binding glucocorticoid receptor (GR) complex in the gene promoter region or by interaction with other transcription factors, particularly activating protein-1 or nuclear factor kappa-β, in the cell nucleus. GR binding to Nf-Kβ loci results in the repression of target genes, thus down-streaming inflammatory signaling [55, 56]. Antimicrobial agents are another topical medication used for the treatment of RAS that protects the ulcer from bacterial infection, yet they exhibit no anti-inflammatory or wound healing promoter effect [2]. There are also wound-healing-promoting agents that neutralize tissue damage from oxidative stress and promote cell proliferation [33]. Liu et al. (2022) recommend using a laser as a short-term alternative intervention during the exacerbation phase of RAS due to its positive effect in accelerating tissue repair and relieving pain [54].

Low-level laser therapy (LLLT) or cold laser is a term used to describe laser applied at an intensity that stimulates biological processes instead of producing ablative or thermal effects. This biological process may produce analgesic and anti-inflammatory effects for pain relief in various diseases [57]. In this review, all 13 trials reporting pain intensity of RAS described reduction after the application of LLLT [12, 14, 33–39, 41–44]. LLLT applied with sufficient intensity inhibits action potentials that cause approximately 30% neural blockade within 10 to 20 min of application and may reverse within about 24 h [58]. Neural blockage results from produced photons that will be absorbed by chromophores in the mitochondria membrane, increasing the production of adenosine triphosphate (ATP) [15, 58]. Adenosine triphosphate (ATP) is the energy source for all cells, and in neurons, this ATP is synthesized by mitochondria located in the dorsal root ganglion. These mitochondria are then transported along the cytoskeleton of the nerve by a monorail system of molecular motors. LLLT has been shown to disrupt the cytoskeleton for hours temporarily, as evidenced by the increase of ATP synthesis, which causes hyperpolarization and stimuli obstruction, thus decreasing pain stimuli induction [58, 59]. The result of this review also synthesized that there is significant difference between group when compared to placebo (sham treatment and motivation only) and topical medication such as glucocorticoid (triamcinolone acetonide 0.1% and betamethasone), antimicrobial agents (dequalinium chloride), wound healing promoting agent (deproteinized calves blood extract 5%), and placebo [12, 14, 33–39, 41–44]. Despite their beneficial effects, topical medication showed limited retention on the targeted site due to salivary flush; therefore, it may contribute to reducing its efficacy for the healing process of an ulcer [2, 10, 54]. Meanwhile, LLLT demonstrates short-term local application in contact or non-contact mode, thus unaffected by salivary wash-out and not presenting with any systemic side effect [15–17].

Four studies investigating the effectivity of LLLT also demonstrated shortened healing time compared to topical medication and placebo. A meta-analysis demonstrated healing time with a mean difference of 2.55 days faster than other therapies (topical medication and placebo) with high heterogeneity (*I*^2^ = 98%) that may result from variability in the type of laser and comparators [20]. In subgroup analysis, the estimated effect of CO2 laser on the reduction of healing time presented a result of 3.72 days faster than placebo (*I*^2^ = 0%). There is strong evidence that LLLT affects the mitochondria of the cells, resulting in the induction of transcription factors that increase the release of growth factors [15]. Low doses of LLLT have been reported to promote cell proliferation of fibroblasts [60, 61] and keratinocytes [62]. Apart from a direct influence on cell proliferation and mitochondrial activity, LLLT also promotes angiogenesis. There is an upregulation of angiogenesis markers VEGF and hypoxia-inducible factor-1α (HIF-1α) with downregulation of tissue remodeling marker matrix metalloproteinase-2 (MMP-2) [63], thus enhancing the proliferation of endothelial cells [64]. Newly formed blood vessels participate in provisional granulation tissue formation, providing nutrition and oxygen to growing tissues [65].

The effectiveness of LLLT depends on many treatment parameters, including wavelength, depth of penetration, size of dose, time of application, level of power density, pulse repetition rate, and treatment protocol [15, 58]. These different parameters all affect the delivered dosage. Dosage measures the energy entering the body and is equal to average power (watts) over treatment time (seconds). The power emitted from the laser probe is determined by the machine's output and is measured in watts. Longer treatment time is associated with a larger dosage of laser administered to the patient [58]. The type of laser included in this review comprised of solid lasers such as Nd: YAG (1064 nm), inert gas lasers such as carbon dioxide (CO2; 10,600 nm), semiconductor laser diodes such as gallium aluminum arsenide (GaAlAs; 809 nm) and InGaAIP (660–670 nm), as well as hydrokinetic laser (Er, Cr: YSGG; 940–2780) [30, 66]. The output power ranged from 0.25 to 1 W, whereas lower output power mostly presented with longer exposure time (varied from 5 to 240 s). The fluency of laser light is mostly in continuous mode at 5–7 mm distance and often pulsed when the laser is in contact with oral mucosa. No agreement has been reported on which continuous wave or pulsed light is better or which factors govern the choice of pulse parameters. Unlike continuous-wave lasers, pulsed lasers dissipate the thermal effect. The pulsed mode has a reduced time of application, but energy levels are still obtained in deeper tissue [58].

Limitations of evidence in this review include reports that resulted in “some concerns” and “high risk” of individual literature bias for not explicitly reporting the randomization process, deviations from the intended interventions, missing outcome data, measurement of the outcome, and selection of the reported result. This affects the confidence of the estimated effect of LLLT in reducing the pain score and healing time of RAS. The limited number of studies also complicates data synthesis because subgroup analysis for each type of laser therapy has yet to be conducted. All studies remained included in data synthesis, considering that the vulnerability to bias resulted from not explicitly reporting the study method instead of bias in study outcomes. Including all eligible studies despite the bias may also provide information for future studies to minimize biases when designing a trial [20, 67].

Despite of reduction in pain score and healing time of RAS after the application of LLLT, clinical applications are inconsistent possibly owing to a lack of comprehension of how dosage is affected by physical and anatomic penetration characteristics [58]. Generalizability in human study is also strenuous as it may be affected by a greater variety of situations and external environments. Future studies may be directed to determine the standardized parameter for low-level laser therapy in recurrent aphthous stomatitis and the type of laser recommended to produce optimum pain reduction and healing in the laboratory setting as it presents greater control of irrelevant variables that might otherwise influence the results of the study [68].

### Supplementary Information

Supplementary Material 1. RoB2.

Supplementary Material 2. Search Strategy.

## Data Availability

All data generated or analyzed during this review are included in this published article and its additional files.
