# Supplementary material for: Effectiveness of low-level laser therapy in reducing pain score and healing time of recurrent aphthous stomatitis: a systematic review and meta-analysis
Source: Syst Rev. 2024 Jul 22;13:192. doi: 10.1186/s13643-024-02595-0 (PMC11264394; doi:10.1186/s13643-024-02595-0)
Supplement: Supplementary file 2 — Supplementary Material 2. Search Strategy. [file 13643_2024_2595_MOESM2_ESM.pdf]

Formulating search statements

(low level laser therapy OR phototherapy laser OR biostimulation laser OR photobiomodulation therapy) AND recurrent aphthous stomatitis.

Population	Patient with recurrent aphthous ulcer
Intervension	Low-level laser therapy
Control	Placebo and medication
Outcome	Reduced pain score and healing time

Database searched: Pubmed, ScienceDirect and Scopus

Date of search: 1 January 1967 to 30 June 2022

Language restriction: English

We contact no individuals or organizations since the information required was assessed based on the article published.

Search strategy was documented in excel as below.

Records marked as ineligible by automation tools based on types of studies. Validated RCT filter were used to exclude study with different design.

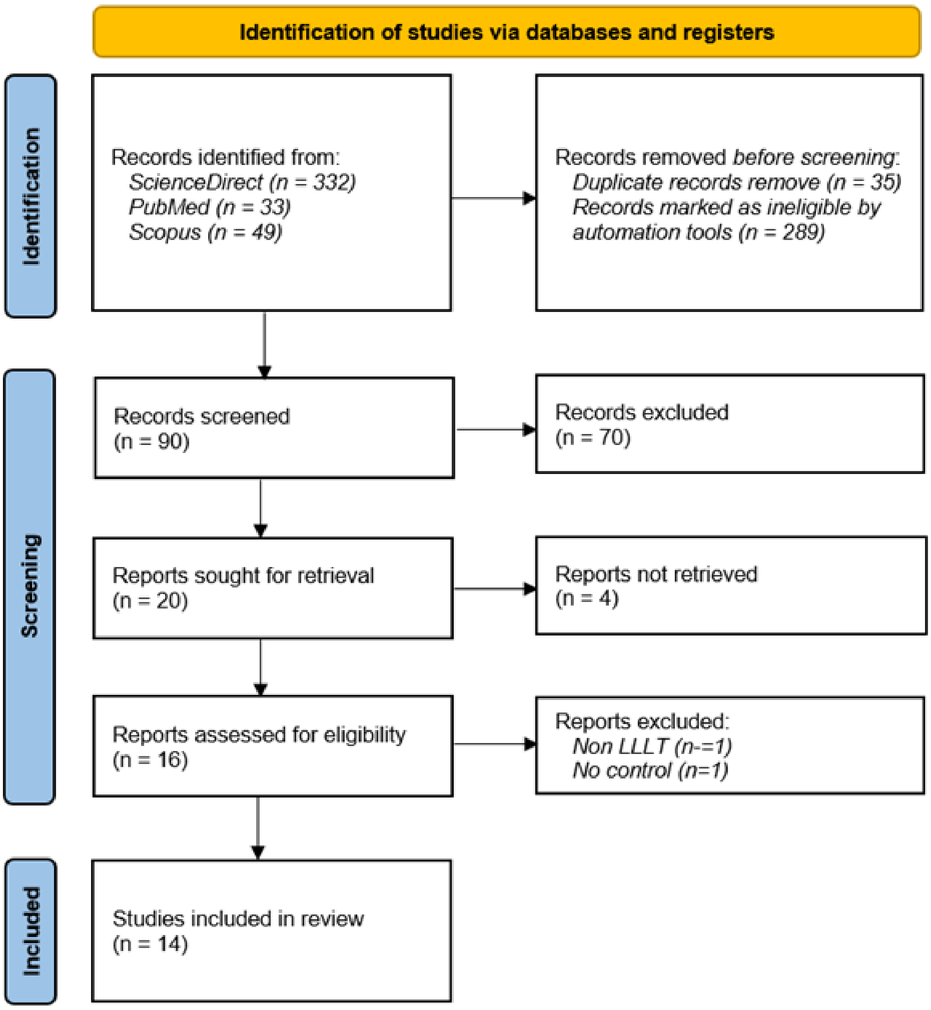

Abstract Excluded	Full-text Excluded	Automatically Removed	Duplicate	Year	Author	Title	Type of Study	Database		
		1		2014	AndrÃ© CV, Bosc	[Low level laser therapy in inflammatory and infectious oral diseases]	Litrev	Pubmed		
				2009	Tezel A, Kara C, E	An evaluation of different treatments for recurrent aphthous stomatitis and patient perceptions: Nd:YAG laser versus medicatior	RCT	Pubmed	1	
			1	2013	Prasad R S, Pai A	Assessment of immediate pain relief with laser treatment in recurrent aphthous stomatitis	RCT	Pubmed		
				2014	Lalabonova H, Da	Clinical assessment of the therapeutic effect of low-level laser therapy on chronic recurrent aphthous stomatitis		Pubmed	1	34
				2019	Soliman HA, Most	Clinical Evaluation of 660 nm Diode Laser Therapy on the Pain, Size and Functional Disorders of Recurrent Aphthous Stomatitis		Pubmed	1	
				2017	Zeini Jahromi N, G	Clinical Evaluation of High and Low-Level Laser Treatment (CO2vsInGaAlP Diode Laser) for Recurrent Aphthous Stomatitis		Pubmed	1	
				2010	De Souza TO, Ma	Clinical evaluation of low-level laser treatment for recurring aphthous stomatitis		Pubmed	1	
				2021	Huo X, Han N, Liu	Effect of different treatments on recurrent aphthous stomatitis: laser versus medication	RCT	Pubmed	1	
			1	2018	Rocca JP, Zhao M	Effect of laser irradiation on aphthae pain management: A four different wavelengths comparison		Pubmed		
		1		2017	Suter VGA, SjÃ¶lu	Effect of laser on pain relief and wound healing of recurrent aphthous stomatitis: a systematic review	Sysrev	Pubmed		
		1		2021	Al-Maweri SA, Ala	Efficacy of hyaluronic acid for recurrent aphthous stomatitis: a systematic review of clinical trials	Sysrev	Pubmed		
		1		2020	Amorim Dos Sant	Laser therapy for recurrent aphthous stomatitis: an overview	Litrev	Pubmed		
		1		2018	Akerzoul N, Chbic	Low laser therapy as an effective treatment of recurrent aphtous ulcers: a clinical case reporting two locations	Case Report	Pubmed		
		1		2013	Anand V, Gulati M	Low level laser therapy in the treatment of aphthous ulcer	Case Report	Pubmed		
			1	2016	Babu DB, Chavva	Low Level Laser Therapy to Reduce Recurrent Oral Ulcers in BehÃ§et's Disease		Pubmed		
			1	2019	Olejnik M, Åšlebio	Low-level laser therapy (LLLT) in the treatment of recurrent aphthous stomatitis (RAS) - a promising treatment option: A report c	Case Report	Pubmed		
		1		2020	Khaleel Ahmed M	Low-Level Laser Therapy and Topical Medications for Treating Aphthous Ulcers: A Systematic Review	Sysrev	Pubmed		
			1	2020	Åšlebioda Z, Dorc	Low-level laser therapy in the treatment of recurrent aphthous stomatitis and oral lichen planus: a literature review	Litrev	Pubmed		
			1	2015	Vale FA, Moreira	Low-level laser therapy in the treatment of recurrent aphthous ulcers: a systematic review	Sysrev	Pubmed		
1				2016	Pentapati KC, Sm	Low-level laser therapy versus 5% amlexanox: a comparison of treatment effects in a cohort of minor aphthous ulcers patients-a commentary		Pubmed		
		1		2016	Spanemberg JC,	Low-level Laser Therapy: A Review of Its Applications in the Management of Oral Mucosal Disorders	Sysrev	Pubmed		
			1	2016	Najeeb S, Khurshi	Management of recurrent aphthous ulcers using low-level lasers: A systematic review	Sysrev	Pubmed		
		1		2019	Kalhori KAM, Vahc	Photobiomodulation in Oral Medicine	Litrev	Pubmed		
		1		2016	Pandeshwar P, Rc	Photobiomodulation in oral medicine: a review	Litrev	Pubmed		
			1	2020	Bardellini E, Vene	Photobiomodulation therapy for the management of recurrent aphthous stomatitis in children: clinical effectiveness and parenta	RCT	Pubmed		
		1		2019	Merigo E, Rocca J	Photobiomodulation Therapy in Oral Medicine: A Guide for the Practitioner with Focus on New Possible Protocols	Litrev	Pubmed		
			1	2012	Zand N, Fateh M,	Promoting wound healing in minor recurrent aphthous stomatitis by non-thermal, non-ablative CO(2) laser therapy: a pilot study	RCT	Pubmed		
			1	2014	Albrektson M, Hec	Recurrent aphthous stomatitis and pain management with low-level laser therapy: a randomized controlled trial	RCT	Pubmed		
				2009	Zand N, Ataie-Fas	Relieving pain in minor aphthous stomatitis by a single session of non-thermal carbon dioxide laser irradiation	RCT	Pubmed	1	
	1			2011	van As G.	The diode laser in treating ulcerative oral lesions		Pubmed		
		1		2015	PavliÄ‡ V, vujiÄ‡-/	Treatment of recurrent aphthous stomatitis by laser therapy: A systematic review of the literature	Sysrev	Pubmed		
			1	2017	Yilmaz HG, Albaba	Treatment of recurrent aphthous stomatitis with Er,Cr:YSGG laser irradiation: A randomized controlled split mouth clinical study	RCT	Pubmed		
				2022	Ghali HGH, Abdul	Treatment of recurrent minor aphthous stomatitis using diode laser (940 nm)	RCT	Pubmed	1	
		1		2012			Book Chapter	ScienceDirect		
		1		2012			Book Chapter	ScienceDirect		
		1		2013			Book Chapter	ScienceDirect		
		1		2009			Book Chapter	ScienceDirect		
		1		2008			Book Chapter	ScienceDirect		
		1		2006			Book Chapter	ScienceDirect		
		1		2006			Book Chapter	ScienceDirect		
		1		2013			Book Chapter	ScienceDirect		
		1		2008			Book Chapter	ScienceDirect		
		1		2003			Book Chapter	ScienceDirect		
		1		2012			Book Chapter	ScienceDirect		
		1		2005			Book Chapter	ScienceDirect		
		1		2008			Book Chapter	ScienceDirect		
		1		2012			Book Chapter	ScienceDirect		
		1		2011			Book Chapter	ScienceDirect		
		1		2011			Book Chapter	ScienceDirect		
		1		2008			Book Chapter	ScienceDirect		
		1		2007			Book Chapter	ScienceDirect		
		1		2016			Book Chapter	ScienceDirect		
		1		2007			Book Chapter	ScienceDirect		
		1		2013			Book Chapter	ScienceDirect		
		1		2012			Book Chapter	ScienceDirect		
		1		2007			Book Chapter	ScienceDirect		
		1		2011			Book Chapter	ScienceDirect		
		1		2013			Book Chapter	ScienceDirect		
		1		2013		10 - How to use the monographs	Book Chapter	ScienceDirect		
		1		2013	Aldred MJ,Came	10 - Paediatric oral medicine, oral pathology and radiology	Book Chapter	ScienceDirect		
		1		2017	Schmidt-Westha	103 - Medical Management of Oral Mucosal Lesions	Book Chapter	ScienceDirect		
		1		2017	Maniar JK,Kamat	11 - HIV and HIV-associated Disorders	Book Chapter	ScienceDirect		
		1		2014	Scully C	11 - Mucosal, oral and cutaneous disorders	Book Chapter	ScienceDirect		
		1		2013	Hallett KB,Alexar	12 - Medically compromised children	Book Chapter	ScienceDirect		
		1		2016	Paller AS,Mancin	15 - Viral Diseases of the Skin	Book Chapter	ScienceDirect		
		1		2011	Paller AS,Mancin	15 - Viral Diseases of the Skin	Book Chapter	ScienceDirect		
		1		2018	Liaqat M,Green J	16 - Aphthous stomatitis	Book Chapter	ScienceDirect		
		1		2018	Watters AL,Hans	16 - Oral/Dental Oncology	Book Chapter	ScienceDirect		
		1		2010	Whitcup SM	19 - Anterior Uveitis	Book Chapter	ScienceDirect		
		1		2011	Paller AS,Mancin	19 - Photosensitivity and Photoreactions	Book Chapter	ScienceDirect		
		1		2016	Paller AS,Mancin	19 - Photosensitivity and Photoreactions	Book Chapter	ScienceDirect		
		1		2019	Flaitz CM	2 - Differential Diagnosis of Oral Lesions and Developmental Anomalies	Book Chapter	ScienceDirect		
		1		2020	Whitcup SM	20 - Anterior Uveitis	Book Chapter	ScienceDirect		
		1		2014	Scully C	20 - Immunodeficiencies	Book Chapter	ScienceDirect		
		1		2022	Bokelmann JM	24 - Aloe (Aloe vera, Aloe barbardensis): Leaf and Leaf Pulp	Book Chapter	ScienceDirect		
		1		2018	Shaw J	25 - Infections of the Oral Cavity	Book Chapter	ScienceDirect		
		1		2018	Eldridge S,Davis I	3.14 - Antineoplastic Agents☆	Book Chapter	ScienceDirect		
		1		2013	Scully C	34 - Aphthae (recurrent aphthous stomatitis)	Book Chapter	ScienceDirect		
		1		2019	Marano AL,Hoot	34 - Cutaneous Disease in Kidney Transplantation Patients	Book Chapter	ScienceDirect		
		1		2005	Vial T,Descotes J,	37 - Drugs that act on the immune system: cytokines and monoclonal antibodies	Book Chapter	ScienceDirect		
		1		2021	Montague L,Clar	4 - Lesions of the Oral Cavity	Book Chapter	ScienceDirect		
		1		2014	Scully C	4 - Signs and symptoms	Book Chapter	ScienceDirect		
		1		2020	Majithia N,Haller	40 - Oral Complications	Book Chapter	ScienceDirect		
		1		2014	Sideras K,Hallem	43 - Oral Complications	Book Chapter	ScienceDirect		
		1		2019	Rosenbaum JT,Li	44 - Ocular, Aural, and Oral Manifestations of Lupus	Book Chapter	ScienceDirect		
		1		2018	Holland KE,Soun	48 - Acquired Rashes in the Older Child	Book Chapter	ScienceDirect		
		1		2019	Gibson JA,Odze F	5 - Tissue Sampling, Specimen Handling, and Laboratory Processing	Book Chapter	ScienceDirect		
1				1997	Khandwala A,Var	5% Amlexanox oral paste, a new treatment for recurrent minor aphthous ulcers: I. Clinical demonstration of accelera	Journal Article	ScienceDirect		
		1		2013	Scully C	54 - Iatrogenic disease	Book Chapter	ScienceDirect		
		1		2008	Kademani D,Bagl	6 - Head and Neck Pathology	Book Chapter	ScienceDirect		
		1		2011	Sawisch TJ	6 - Oral Surgery for the General Practitioner	Book Chapter	ScienceDirect		
		1		2021	Schlosser BJ,Dup	61 - Oral Mucosal Therapeutics	Book Chapter	ScienceDirect		
		1		2018	Johnson MM	7 - Ear, Nose, and Throat Infections	Book Chapter	ScienceDirect		
		1		2014	Scully C	7 - Gastrointestinal and pancreatic disorders	Book Chapter	ScienceDirect		
		1		2019	Neville BW,Damr	7 - Viral Infections	Book Chapter	ScienceDirect		
		1		2014	Scully C	8 - Haematology	Book Chapter	ScienceDirect		
		1		2019	Neville BW,Damr	8 - Physical and Chemical Injuries	Book Chapter	ScienceDirect		
		1		2013	Kim S,Michaels B	8 - Systemic Antibacterial Agents	Book Chapter	ScienceDirect		
		1		2010	Weedon D	8 - The vasculopathic reaction pattern	Book Chapter	ScienceDirect		
		1		2016	Sawisch TJ,Deeb	8 - Use of Lasers for Minor Oral Surgery in General Practice	Book Chapter	ScienceDirect		

		1		2013		9 - Herbal approaches to system dysfunctions	Book Chapter	ScienceDirect	
		1		2021	Snodgrass A,Mot	9 - Systemic Antibacterial Agents	Book Chapter	ScienceDirect	
		1		2021		A	Book Chapter	ScienceDirect	
		1		2014		A	Book Chapter	ScienceDirect	
		1		2007		A	Book Chapter	ScienceDirect	
1				2017	Moghadam-Kia S	A diagnostic and therapeutic approach to primary burning mouth syndrome	Journal Article	ScienceDirect	
1				1993	Greer RO,Linden	A double-blind study of topically applied 5% amlexanox in the treatment of aphthous ulcers	Journal Article	ScienceDirect	
1				2012	Meiller TF,Garbe	A Review of Common Oral Pathology Lesions, With a Focus on Periodontology and Implantology	Journal Article	ScienceDirect	
		1		2005		ABSTRACT BOOK	Journal Article	ScienceDirect	
		1		2013		Abstract Book	Journal Article	ScienceDirect	
		1		1999		Abstracts	Journal Article	ScienceDirect	
		1		2010		Abstracts	Journal Article	ScienceDirect	
		1		1989		Abstracts	Journal Article	ScienceDirect	
		1		1998		Abstracts of papers presented at the 52nd annual meeting of the American Academy of Oral Medicine	Journal Article	ScienceDirect	
		1		1994		Abstracts of papers presented at the American Academy of Oral Medicine Annual Meeting, Clearwater, Fla., April 27-	Journal Article	ScienceDirect	
1				2022	Patil S,Mustaq S,	Advancement in therapeutic strategies for immune-mediated oral diseases	Journal Article	ScienceDirect	
1				2009	Parent D,Vaillant	Afte, aftosi, malattia di Behçet	Journal Article	ScienceDirect	
		1		2002		AGA abstracts 82-724	Journal Article	ScienceDirect	
		1		2002		AGA abstracts T1590-W1053	Journal Article	ScienceDirect	
1				2000	Halpern LR,Ogle	Alternative Treatment Modalities for Orofacial Pain	Journal Article	ScienceDirect	
		1		2019	Cagna DR,Donov	Annual review of selected scientific literature: A report of the Committee on Scientific Investigation of the American A	Journal Article	ScienceDirect	
		1		2021	Cagna DR,Donov	Annual review of selected scientific literature: A report of the Committee on Scientific Investigation of the American A	Journal Article	ScienceDirect	
		1		2000	Sadick NS	Antibiotics: unapproved uses or indications	Journal Article	ScienceDirect	
		1		2016	Vaillant L,Samimi	Aphtes et ulcérations buccales	Journal Article	ScienceDirect	
		1		2016		Aphthous Stomatitis	Book Chapter	ScienceDirect	
		1		2007	Boulanger BL	Aphthous Ulcers	Book Chapter	ScienceDirect	
		1		2021		Appendix - Supplemental Material	Book Chapter	ScienceDirect	
				2013	Prasad R. S,Pai A	Assessment of immediate pain relief with laser treatment in recurrent aphthous stomatitis	Journal Article	ScienceDirect	1
		1		1997		Author index	Journal Article	ScienceDirect	
		1		1994		Author index	Journal Article	ScienceDirect	
		1		1996		Author index	Journal Article	ScienceDirect	
		1		1988		Author index	Journal Article	ScienceDirect	
		1		1997		Author index	Journal Article	ScienceDirect	
		1		1996		Author index	Journal Article	ScienceDirect	
		1		2011	Sciubba JJ	Autoimmune Oral Mucosal Diseases: Clinical, Etiologic, Diagnostic, and Treatment Considerations	Journal Article	ScienceDirect	
		1		2013		Auto-Immunity & Inflammation	Journal Article	ScienceDirect	
		1		2007	Lachman RS	B	Book Chapter	ScienceDirect	
1				2017	Bulur I,Onder M	Behçet disease: New aspects	Journal Article	ScienceDirect	
		1		2012	Chan MH,Wolf JC	Biopsy Techniques and Diagnoses & Treatment of Mucocutaneous Lesions	Journal Article	ScienceDirect	
		1		2003	Drage LA,Rogers	Burning mouth syndrome	Journal Article	ScienceDirect	
		1		2015	Balakumar P,Kav	Cardiovascular drugs-induced oral toxicities: A murky area to be revisited and illuminated	Journal Article	ScienceDirect	
		1		2009	Burris K,Patel G,L	Chapter 10 - Diseases of the mouth and oral mucosa	Book Chapter	ScienceDirect	
		1		2006	Maniar J,Kamath	Chapter 11 - HIV and HIV-associated disorders	Book Chapter	ScienceDirect	
		1		2007	Crum PM,DesRos	CHAPTER 11 - Oral Cancer: Prevention, Management, and Treatment	Book Chapter	ScienceDirect	
		1		2004	Sapp JP,Eversole	CHAPTER 12 - Diseases of Blood	Book Chapter	ScienceDirect	
		1		2009	Skvarka CB,Ko CJ	Chapter 13 - Lichenoid dermatoses	Book Chapter	ScienceDirect	
		1		2016	John V,Weddell J	Chapter 14 - Gingivitis and Periodontal Disease	Book Chapter	ScienceDirect	
		1		2009	Weidner N,Matth	CHAPTER 14 - Oral Cavity and Jaws	Book Chapter	ScienceDirect	
		1		2008	Elad S,Epstein J,K	Chapter 14 - Orofacial pain in the medically complex patient	Book Chapter	ScienceDirect	
		1		2007	Wetmore RF	CHAPTER 15 - Approach to the Pediatric Neck Mass	Book Chapter	ScienceDirect	
		1		2020	Pansare R,Nagas	Chapter 15 - Dermatological Toxicities of Targeted Therapy	Book Chapter	ScienceDirect	
		1		2012	Maurer T,Michel	Chapter 17 - Global HIV and dermatology	Book Chapter	ScienceDirect	
		1		2009	Benoliel R,Pertes	Chapter 17 - OROFACIAL PAIN	Book Chapter	ScienceDirect	
		1		2013	Little JW,Falace D	Chapter 18 - AIDS, HIV Infection, and Related Conditions	Book Chapter	ScienceDirect	
		1		2012	O'Handley JG,To	Chapter 19 - Otorhinolaryngology	Book Chapter	ScienceDirect	
		1		2011	McDonald RE,Av	CHAPTER 20 - Gingivitis and Periodontal Disease	Book Chapter	ScienceDirect	
		1		2012	Liu JK,Lichtenstei	Chapter 21 - Inflammatory Bowel Disease	Book Chapter	ScienceDirect	
		1		2008	Maurer T	CHAPTER 22 - Global HIV and Dermatology	Book Chapter	ScienceDirect	
		1		2021	Jung L,Paul WM	Chapter 23 - Autoimmune disorders	Book Chapter	ScienceDirect	
		1		2009	Petras RE,Franke	CHAPTER 23 - Large Intestine (Colon)	Book Chapter	ScienceDirect	
		1		2011	Sanders BJ,Shapi	CHAPTER 24 - Management of the Medically Compromised Patient: Hematologic Disorders, Cancer, Hepatitis, and AII	Book Chapter	ScienceDirect	
		1		2009	Herron MD,Zone	Chapter 25 - Cutaneous Diseases Associated with Gastrointestinal Abnormalities	Book Chapter	ScienceDirect	
		1		2004	Sanchez M,Fried	Chapter 25 - Skin Manifestations of HIV Infection	Book Chapter	ScienceDirect	
		1		2016	Sanders BJ,Shapi	Chapter 26 - Management of the Medically Compromised Patient: Hematologic Disorders, Cancer, Hepatitis, and AID	Book Chapter	ScienceDirect	
		1		2021	Concha JS,Werth	Chapter 28 - Skin	Book Chapter	ScienceDirect	
		1		2007	Scully C,Diz Dios	Chapter 3 - SPECIFIC PROBLEM AREAS	Book Chapter	ScienceDirect	
		1		2012		Chapter 3 - White Lesions	Book Chapter	ScienceDirect	
		1		2014	Lally A,Jenkins SN	Chapter 34 - Non-Malignant and Malignant Skin Lesions in Kidney Transplant Patients	Book Chapter	ScienceDirect	
		1		2012		Chapter 36 - Chronic Disease Management	Book Chapter	ScienceDirect	
		1		2009	Bouquot JE,Mulle	Chapter 4 - Lesions of the Oral Cavity	Book Chapter	ScienceDirect	
		1		2017	Franz-Montan M	Chapter 4 - Nanostructured systems for transbuccal drug delivery	Book Chapter	ScienceDirect	
		1		2011	Werth VP,Vera K	Chapter 41 - Skin	Book Chapter	ScienceDirect	
		1		2009	Chacko MR,Staat	CHAPTER 48 - GENITAL INFECTIONS	Book Chapter	ScienceDirect	
		1		2012	Elden LM,Wetmo	Chapter 55 - Otolaryngologic Disorders	Book Chapter	ScienceDirect	
		1		2004	Nopper AJ,Georg	Chapter 56 - Rashes and Skin Lesions	Book Chapter	ScienceDirect	
		1		2010	Johnson MM	Chapter 6 - Ear, Nose, and Throat Infections	Book Chapter	ScienceDirect	
		1		2014		Chapter 7 - Head and Neck Pathology	Book Chapter	ScienceDirect	
		1		2004	Sapp JP,Eversole	CHAPTER 7 - Oral Infections	Book Chapter	ScienceDirect	
		1		2020	Sandri G,Ruggeri	Chapter 8 - (Trans)buccal drug delivery	Book Chapter	ScienceDirect	
		1		2006		Chapter Four - Pathology	Book Chapter	ScienceDirect	
		1		2020	Xerez MC,Costa C	CLINICAL AND SURGICAL CONDUCT IN AMELOBLASTOMA OF LARGE DIMENSIONS	Journal Article	ScienceDirect	
		1		2018	Davis MD,Litin SC	Clinical pearls in dermatology 2018	Journal Article	ScienceDirect	
		1		2004	Sharon-Buller A,S	CO2-laser treatment of ulcerative lesions	Journal Article	ScienceDirect	
1				1990	Halvorsen JG	Common Problems of the Oral Cavity	Journal Article	ScienceDirect	
		1		2009		Communications orales	Journal Article	ScienceDirect	
1				2020	John SS,Mohanty	Comparative evaluation of Low Level Laser Therapy and cryotherapy in pain control and wound healing following ort	Journal Article	ScienceDirect	
1				2006	Beltrani VS,Berns	Contact dermatitis: a practice parameter	Journal Article	ScienceDirect	
		1		2010		Contents	Journal Article	ScienceDirect	
		1		2014		Contents: Volume 42	Journal Article	ScienceDirect	
		1		2005		Cumulative Index	Journal Article	ScienceDirect	
		1		1990		Cumulative Index 1990	Journal Article	ScienceDirect	
		1		1991		Cumulative Index 1991	Journal Article	ScienceDirect	
		1		1992		CUMULATIVE INDEX 1992	Journal Article	ScienceDirect	
		1		2000		CUMULATIVE INDEX 2000	Journal Article	ScienceDirect	
		1		2004		Cumulative Index 2004	Journal Article	ScienceDirect	
		1		2005	Evereklioglu C	Current Concepts in the Etiology and Treatment of Behçet Disease	Journal Article	ScienceDirect	
		1		2020	Moghadam ET,Ya	Current herbal medicine as an alternative treatment in dentistry: In vitro, in vivo and clinical studies	Journal Article	ScienceDirect	
		1		1987		Current papers in Oral Biology (34622–34725)	Journal Article	ScienceDirect	
		1		1994	Prussick R,Knowl	Cutaneous drug reactions	Journal Article	ScienceDirect	
		1		2013	Thrash B,Patel M	Cutaneous manifestations of gastrointestinal disease: Part II	Journal Article	ScienceDirect	
1				2021	Mármora BC,Bro	Defocused high-power diode laser accelerates skin repair in a murine model through REDOX state modulation and re	Journal Article	ScienceDirect	
1				1993	Coskey RJ	Dermatologic therapy: 1992	Journal Article	ScienceDirect	
1				1985	Coskey RJ	Dermatologic therapy: December 1983 through November 1984	Journal Article	ScienceDirect	

1				1982	Coskey RJ	Dermatologic therapy: December, 1980, through November, 1981	Journal Article	ScienceDirect
1				1998	Barr CE,Glick M	Diagnosis and Management of Oral and Cutaneous Lesions in HIV-1 Disease	Journal Article	ScienceDirect
1				1998	Reich RF,Kerpel S	Differential Diagnosis and Treatment of Ulcerative, Erosive, and Vesiculobullous Lesions of the Oral Mucosa	Journal Article	ScienceDirect
1				2017	Greenberg SA,Sc	Diseases of the lips	Journal Article	ScienceDirect
		1		1996	Camisa C,Rindler	Diseases of the oral mucous membranes	Journal Article	ScienceDirect
1				2020	Iglesias-Sancho N	Drug Compounding for Diseases of the Oral Mucosa	Journal Article	ScienceDirect
1				2001	Shin HT,Chang M	Drug eruptions in children	Journal Article	ScienceDirect
1				2002	Shin HT,Change I	Drug eruptions in children	Journal Article	ScienceDirect
		1		2011		ECCO Congress Abstracts	Journal Article	ScienceDirect
				2018	Rocca JP,Zhao M	Effect of laser irradiation on aphthae pain management: A four different wavelengths comparison	Journal Article	ScienceDirect
		1		2019	Bhattacharya PT,	Effectiveness of 904nm Gallium-Arsenide Diode Laser in Treatment of Oral Lichen Planus: Report of 2Cases	Journal Article	ScienceDirect
1				2013	Jiang XW,Zhang Y	Effects of berberine gelatin on recurrent aphthous stomatitis: a randomized, placebo-controlled, double-blind trial in	Journal Article	ScienceDirect
		1		2017	Al-Maweri SA,Jav	Efficacy of low level laser therapy in the treatment of burning mouth syndrome: A systematic review	Journal Article	ScienceDirect
1				2014	Levy-Clarke G,Jab	Expert Panel Recommendations for the Use of Anti–Tumor Necrosis Factor Biologic Agents in Patients with Ocular Inf	Journal Article	ScienceDirect
		1		2020	Netto JD,Mirand	EXUBERANT SYMMETRIC LIPOMATOSIS OF THE TONGUE	Journal Article	ScienceDirect
		1		2017	Chusid MJ	Fever of Unknown Origin in Childhood	Journal Article	ScienceDirect
		1		2018	World Dental Fe	Friday, 7 September 2018 - Free Communication Sessions 28–42 and Poster Sessions 31–45	Journal Article	ScienceDirect
		1		2015	World Dental Fe	Friday, September 12, 2014 - FREE COMMUNICATIONS SESSIONS 05-08	Journal Article	ScienceDirect
1				2018	Matthews N,Wo	Genital diseases in the mature woman	Journal Article	ScienceDirect
		1		2005	Lyon KF	Gingivostomatitis	Journal Article	ScienceDirect
1				1997	Task Force:,Rico	Guidelines of care for dermatologic conditions in patients infected with HIV	Journal Article	ScienceDirect
1				1997	Chairman MJ,My	Guidelines of care for dermatologic conditions in patients infected with HIV	Journal Article	ScienceDirect
		1		2019	Kao JH	Highlights	Journal Article	ScienceDirect
		1		2008	Smith JA	HIV and AIDS in the Adolescent and Adult: An Update for the Oral and Maxillofacial Surgeon	Journal Article	ScienceDirect
		1		2003	Patton LL	HIV Disease	Journal Article	ScienceDirect
1				1994	Porter SR,Scully C	HIV: The surgeon's perspective Part 2. Diagnosis and management of non-malignant oral manifestations	Journal Article	ScienceDirect
		1		2022	Besegato JF,de M	How can biophotonics help dentistry to avoid or minimize cross infection by SARS-CoV-2?	Journal Article	ScienceDirect
		1		2007	Fatahzadeh M,Sc	Human herpes simplex virus infections: Epidemiology, pathogenesis, symptomatology, diagnosis, and management	Journal Article	ScienceDirect
		1		2001	Ramsay A,Lightm	Hypopyon Uveitis	Journal Article	ScienceDirect
1				2016	Fellmann F,Ange	IL-17 receptor A and adenosine deaminase 2 deficiency in siblings with recurrent infections and chronic inflammatio	Journal Article	ScienceDirect
		1		2020		Index	Book Chapter	ScienceDirect
		1		2015		Index	Book Chapter	ScienceDirect
		1		2017		Index	Book Chapter	ScienceDirect
		1		2018		Index	Book Chapter	ScienceDirect
		1		2015		Index	Book Chapter	ScienceDirect
		1		2017		Index	Book Chapter	ScienceDirect
		1		2019		Index	Book Chapter	ScienceDirect
		1		2020		Index	Book Chapter	ScienceDirect
		1		2016		Index	Book Chapter	ScienceDirect
		1		2013		Index	Book Chapter	ScienceDirect
		1		2018		Index	Book Chapter	ScienceDirect
		1		2016		Index	Book Chapter	ScienceDirect
		1		2014		Index	Book Chapter	ScienceDirect
		1		2020		Index	Book Chapter	ScienceDirect
		1		2015		Index	Book Chapter	ScienceDirect
		1		2018		Index	Book Chapter	ScienceDirect
		1		2021		Index	Book Chapter	ScienceDirect
		1		1980		Index	Journal Article	ScienceDirect
		1		2014		Index of drugs	Book Chapter	ScienceDirect
		1		1990		Index to volume 11	Journal Article	ScienceDirect
		1		1996		Index to volume 34 subject index	Journal Article	ScienceDirect
		1		2004		Index to volume 50	Journal Article	ScienceDirect
		1		2004		Index to volume 50	Journal Article	ScienceDirect
		1		1989		INDEX TO VOLUME I, 1989	Journal Article	ScienceDirect
1				1999	Bordon E,Bordon	INFECTIOUS DISEASE CONCERNS AND POSSIBLE COMPLICATIONS IN THE DENTAL PATIENT	Journal Article	ScienceDirect
1				1987	Lawson W,Blitzer	Inflammatory and neoplastic lesions of the oral cavity	Journal Article	ScienceDirect
			1	2016	Gutiérrez-Vargas	Instruments to measure the quality of life in patients with oral mucositis undergoing oncological treatment: a system	Journal Article	ScienceDirect
1				2016	Gutiérrez-Vargas	Instruments to measure the quality of life in patients with oral mucositis undergoing oncological treatment: a system	Journal Article	ScienceDirect
1				2014	Uberti G,Goldblu	Ischemic enterocolitis and its differential diagnosis	Journal Article	ScienceDirect
		1		2003	Eguía A,Saldón R	La Estomatitis Aftosa Recurrente (II): Aspectos diagnósticos y terapéuticos	Journal Article	ScienceDirect
		1		2017	Brignardello-Pete	Laser use may improve pain and wound healing in patients with recurrent aphtous stomatitis	Journal Article	ScienceDirect
	1			2008	Kotlow L	Lasers and Soft Tissue Treatments for the Pediatric Dental Patient	Journal Article	ScienceDirect
1				2000	Convissar RA	LASERS IN A HOSPITAL-BASED DENTAL PRACTICE	Journal Article	ScienceDirect
		1		2004	Convissar RA	Lasers in general dentistry	Journal Article	ScienceDirect
		1		2017		Liste des abréviations	Book Chapter	ScienceDirect
1				2010	Robertson SA,La	Long-Term pain in cats: How much do we know about this important welfare issue?	Journal Article	ScienceDirect
		1		2019	Jácome-Santos H	Low-level laser as a complementary therapy in orofacial granulomatosis management: a case report	Journal Article	ScienceDirect
			1	2016	Jijin MJ,Rakarad	Low-level laser therapy versus 5% amlexanox: a comparison of treatment effects in a cohort of patients with minor a	Journal Article	ScienceDirect
1				1992	Scully C,McCarth	Management of oral health in persons with HIV infection	Journal Article	ScienceDirect
			1	2016	Najeeb S,Khurshi	Management of recurrent aphthous ulcers using low-level lasers: A systematic review	Journal Article	ScienceDirect
1				2015	Landry BW,Fisch	Managing Chronic Pain in Children and Adolescents: A Clinical Review	Journal Article	ScienceDirect
		1		1991	van Riel PL	Metals	Book Chapter	ScienceDirect
1		1		2020	Sganzerla JT,Kru	MOUTH ULCERS IN CANCER PATIENTS UNDERGOING EXPERIMENTAL TREATMENT WITH IMMUNE CHECKPOINT INHIB	Journal Article	ScienceDirect
		1		2016	De Sanctis V,Bos	Mucositis in head and neck cancer patients treated with radiotherapy and systemic therapies: Literature review and	Journal Article	ScienceDirect
1				1987	Case JD,Callen JP	Mucous membranes in systemic disease	Journal Article	ScienceDirect
1				2016	Wanat KA,Kim B,	Multisystem diseases affecting the skin and eye	Journal Article	ScienceDirect
1				2020	Kia SJ,Mansouria	New concentration of curcumin orabase in recurrent aphthous stomatitis: A randomized, controlled clinical trial	Journal Article	ScienceDirect
		1		2014	Sehgal VN,Pandh	Nonspecific genital ulcers	Journal Article	ScienceDirect
		1		2020	Silva WR,França	ODONTOGENIC FIBROMIXOMA: A CASE REPORT OF AN UNCOMMON NEOPLASM	Journal Article	ScienceDirect
		1		2020	Santana LA,Aguia	ODONTOMA-ASSOCIATED CALCIFYING ODONTOGENIC CYST MIMETIZING DENTAL GERM	Journal Article	ScienceDirect
		1		2008	Guhl G,Díaz-Ley	Off-Label Use of Biologic Agents in the Treatment of Dermatosis, Part 2: Etanercept, Efalizumab, Alefacept, Rituximab	Journal Article	ScienceDirect
		1		2022	Tuchin VV,Genin	Optical clearing of tissues: Issues of antimicrobial phototherapy and drug delivery	Journal Article	ScienceDirect
		1		1996	Singh N,Scully C,	Oral complications of cancer therapies: Prevention and management	Journal Article	ScienceDirect
		1		2009	Leao JC,Ribeiro C	Oral Complications of HIV Disease	Journal Article	ScienceDirect
		1		2017	Elad S,Zadik Y,Ya	Oral Complications of Nonsurgical Cancer Therapies	Journal Article	ScienceDirect
		1		2000	Tüzün B,Tüzün Y	Oral disorders: unapproved treatments or indications11The opinions expressed herein are solely those of the investi	Journal Article	ScienceDirect
		1		2008	Schubert MM,Co	Oral Graft-Versus-Host Disease	Journal Article	ScienceDirect
1				2010	Warnakulasuriya	Oral health risks of tobacco use and effects of cessation	Journal Article	ScienceDirect
		1		2021	Asan MF,Castelin	Oral Immune-Related Adverse Events – Current Concepts and their Management	Journal Article	ScienceDirect
1				1999	Patton LL,van de	ORAL INFECTIONS AND OTHER MANIFESTATIONS OF HIV DISEASE	Journal Article	ScienceDirect
		1		2013	Patton LL	Oral Lesions Associated with Human Immunodeficiency Virus Disease	Journal Article	ScienceDirect
		1		2003	Patel NJ,Sciubba	Oral lesions in young children	Journal Article	ScienceDirect
		1		1993	Itin PH,Lautensch	Oral manifestations in HIV-infected patients: Diagnosis and management	Journal Article	ScienceDirect
1				2014	Cowan GM,Locke	Oral Manifestations of Allergic, Infectious, and Immune-mediated Disease	Journal Article	ScienceDirect
		1		2017	Mejia LM	Oral Manifestations of Gastrointestinal Disorders	Journal Article	ScienceDirect
		1		2000	Casiglia JW,Woo	Oral manifestations of HIV infection	Journal Article	ScienceDirect
1				1991	Scully C,Laskaris	Oral manifestations of HIV infection and their management. I. More common lesions	Journal Article	ScienceDirect
		1		2017	Johnson L,Persch	Oral Manifestations of Immunologically Mediated Diseases	Journal Article	ScienceDirect
		1		2014	Stoopler ET,Solle	Oral Mucosal Diseases: Evaluation and Management	Journal Article	ScienceDirect
		1		2005	Lalla RV,Peterson	Oral mucositis	Journal Article	ScienceDirect
1				2022	Shetty SS,Maruth	Oral mucositis: Current knowledge and future directions	Journal Article	ScienceDirect
		1		2011	Sanjar FA,Queiro	Otolaryngologic manifestations in HIV disease - clinical aspects and treatment	Journal Article	ScienceDirect
1				1999	Chandrasekhar J	Oxypentifylline in the management of recurrent aphthous oral ulcersAn open clinical trial	Journal Article	ScienceDirect

		1		2011	Ferri FF	P	Book Chapter	ScienceDirect	
		1		1980	Rees RB,Odom R	Pacific dermatologic association: Thirty-first Annual Meeting, San Francisco, CA, Sept. 16–20, 1979	Journal Article	ScienceDirect	
		1		2014	Sardana K,Bansa	Palatal ulceration	Journal Article	ScienceDirect	
		1		2003	Witman PM,Roge	Pediatric oral medicine	Journal Article	ScienceDirect	
1				2000	Delaney JE,Keels	PEDIATRIC ORAL PATHOLOGY: Soft Tissue and Periodontal Conditions	Journal Article	ScienceDirect	
		1		2016		Penicillamine	Book Chapter	ScienceDirect	
1				2020	Convissar RA,Ros	Photobiomodulation lasers in dentistry	Journal Article	ScienceDirect	
1				2014	André CV,Bosc R	Photothérapie par diode électroluminescente des pathologies inflammatoires et infectieuses de la cavité orale	Journal Article	ScienceDirect	
		1		1996	Kassan DG,Lynch	Physical enhancement of dermatologic drug delivery: Iontophoresis and phonophoresis	Journal Article	ScienceDirect	
		1		2021	Peranzzetta TS,C	PLASMA CELL CHEILITIS: REPORT OF 2 CASES FROM BRAZIL	Journal Article	ScienceDirect	
		1		2009		Poster Sessions	Journal Article	ScienceDirect	
		1		2008		Posters	Journal Article	ScienceDirect	
1				1994	Barr CE	PRACTICAL CONSIDERATIONS IN THE TREATMENT OF THE HIV-INFECTED PATIENT	Journal Article	ScienceDirect	
		1		2022	Abiko Y,Paudel D	Psychostomatology: The psychosomatic status and approaches for the management of patients with inflammatory o	Journal Article	ScienceDirect	
1				1998	Le Cleach L,Bocq	REACTIONS AND INTERACTIONS OF SOME COMMONLY USED SYSTEMIC DRUGS IN DERMATOLOGY	Journal Article	ScienceDirect	
1				2016	Cui RZ,Bruce AJ,R	Recurrent aphthous stomatitis	Journal Article	ScienceDirect	
		1		2012		Recurrent aphthous stomatitis	Journal Article	ScienceDirect	
		1		2020	Sánchez J,Coneje	Recurrent Aphthous Stomatitis	Journal Article	ScienceDirect	
1				2000	Porter SR,Hegart	Recurrent aphthous stomatitis	Journal Article	ScienceDirect	
				2014	Albrektson M,He	Recurrent aphthous stomatitis and pain management with low-level laser therapy: a randomized controlled trial	Journal Article	ScienceDirect	1
		1		1996	Ship JA	Recurrent aphthous stomatitis: An update	Journal Article	ScienceDirect	
1				2018	Queiroz SI,da Silv	Recurrent aphthous ulceration: an epidemiological study of etiological factors, treatment and differential diagnosis*	Journal Article	ScienceDirect	
1				1996	Woo SB,Sonis ST	RECURRENT APHTHOUS ULCERS: A REVIEW OF DIAGNOSIS AND TREATMENT	Journal Article	ScienceDirect	
		1		2015	World Dental Fe	Saturday, September 13, 2014 - FREE COMMUNICATIONS SESSIONS 09-12	Journal Article	ScienceDirect	
		1		2008		Scientific Abstract Sessions	Journal Article	ScienceDirect	
		1		2015		Scientific Abstract Sessions	Journal Article	ScienceDirect	
		1		2021	Bashir NZ,Carate	Silver nitrate in the management of recurrent aphthous stomatitis: A systematic review and meta-analysis	Journal Article	ScienceDirect	
		1		1999	Camilleri MJ,Calc	Skin eruptions in the diaper area	Journal Article	ScienceDirect	
1				1991	Rice DH	Snoring and Obstructive Sleep Apnea	Journal Article	ScienceDirect	
		1		2017	Wilder EG,Friede	Spectrum of orocutaneous disease associations: Genodermatoses and inflammatory conditions	Journal Article	ScienceDirect	
		1		2004		Subject Index	Book Chapter	ScienceDirect	
		1		1994		Subject index	Journal Article	ScienceDirect	
		1		1997		Subject index	Journal Article	ScienceDirect	
		1		1996		Subject index	Journal Article	ScienceDirect	
		1		1991		Subject index	Journal Article	ScienceDirect	
		1		2005		Subject Index	Journal Article	ScienceDirect	
		1		1996		Subject index	Journal Article	ScienceDirect	
		1		1997		Subject index	Journal Article	ScienceDirect	
		1		2012		Subject index	Journal Article	ScienceDirect	
		1		2000		Subject index	Journal Article	ScienceDirect	
		1		1998		Subject index	Journal Article	ScienceDirect	
		1		2016		Subject Index: Annual Index 2016	Journal Article	ScienceDirect	
		1		2003		SUBJECT INDEX: VOLUME 134, 2003	Journal Article	ScienceDirect	
		1		2022	Kalogeropoulos	The association between intestinal microbiome and autoimmune uveitis	Journal Article	ScienceDirect	
1				2005	Materia E,Baglio	The clinical and organisational appropriateness of tonsillectomy and adenoidectomy—an Italian perspective	Journal Article	ScienceDirect	
		1		2016	Radulescu M	The Pharmacologic Management of Common Lesions of the Oral Cavity	Journal Article	ScienceDirect	
1				2001	Ogle OE,Ofodile	The Pharmacology of Topical Agents	Journal Article	ScienceDirect	
		1		2014		Therapeutic Management of Common Oral Lesions: Based on Material from the American Academy of Oral Medicine	Book Chapter	ScienceDirect	
		1		2017	World Dental Fe	Thursday, 31 August 2017 - Free Communication Sessions 49–72 and Poster Sessions 48–67	Journal Article	ScienceDirect	
		1		2011	Chiu HY,Tsai TF	Topical use of systemic drugs in dermatology: A comprehensive review	Journal Article	ScienceDirect	
				2017	Yilmaz HG,Albab	Treatment of recurrent aphthous stomatitis with Er,Cr:YSGG laser irradiation: A randomized controlled split mouth c	Journal Article	ScienceDirect	1
		1		2017	World Dental Fe	Tuesday, 29 August 2017 – Free Communication Sessions 01–24 and Poster Sessions 01–24	Journal Article	ScienceDirect	
1				2000	Stone SP	UNUSUAL, INNOVATIVE, AND LONG-FORGOTTEN REMEDIES	Journal Article	ScienceDirect	
		1		1999	Severson JL,Tyrin	Viral disease update	Journal Article	ScienceDirect	
		1		2007		Volume 52, 2007 Contents and Author Index	Journal Article	ScienceDirect	
		1		2002	Foster DC	Vulvar disease	Journal Article	ScienceDirect	
		1		1997		Washington DC	Journal Article	ScienceDirect	
		1		2016	World Dental Fe	Wednesday, 7 September 2016 - AWDC 2016 – ABSTRACT BOOK - FREE COMMUNICATION SESSIONS 01–18 POSTER S	Journal Article	ScienceDirect	
	1			2013	Misra N,Maiti D,	940 nm diode laser therapy in management of recurrent apthous ulcer	Journal Article	Scopus	
		1		2019	Banu S,Ramakris	A comprehensive review on oral aphthous ulcer	Journal Article	Scopus	
				2018	Mustafa NS,Kash	A pilot study on the use of biolase in the treatment of recurrent aphthous ulcer	Journal Article	Scopus	1
			1	2009	Tezel A,Kara C,Ba	An evaluation of different treatments for recurrent aphthous stomatitis and patient perceptions: Nd:YAG laser versu	Journal Article	Scopus	
			1	2013	Prasad R. S,Pai A	Assessment of immediate pain relief with laser treatment in recurrent aphthous stomatitis	Journal Article	Scopus	
		1		2019	Akdeniz N,Elmas	Behçet syndrome: A great imitator	Journal Article	Scopus	
			1	2014	Lalabonova H,Da	Clinical assessment of the therapeutic effect of low-level laser therapy on chronic recurrent aphthous stomatitis	Journal Article	Scopus	
			1	2019	Soliman HA,Mos	Clinical evaluation of 660 nm diode laser therapy on the pain, size and functional disorders of recurrent aphthous sto	Journal Article	Scopus	
			1	2010	De Souza TO,Ma	Clinical evaluation of low-level laser treatment for recurring aphthous stomatitis	Journal Article	Scopus	
			1	2021	Huo X,Han N,Liu	Effect of different treatments on recurrent aphthous stomatitis: laser versus medication	Journal Article	Scopus	
			1	2018	Rocca JP,Zhao M	Effect of laser irradiation on aphthae pain management: A four different wavelengths comparison	Journal Article	Scopus	
			1	2017	Suter VG,Sjölund	Effect of laser on pain relief and wound healing of recurrent aphthous stomatitis: a systematic review	Journal Article	Scopus	
			1	2021	Al-Maweri SA,Ala	Efficacy of hyaluronic acid for recurrent aphthous stomatitis: a systematic review of clinical trials	Journal Article	Scopus	
		1		2020	Navabi N,Kamiab	Etiology and treatment of oral recurrent aphthous stomatitis	Journal Article	Scopus	
		1		2009	Demetriades N,H	General manifestations of Behçet's syndrome and the success of CO2-laser as treatment for oral lesions: a review of	Journal Article	Scopus	
			1	2020	Amorim dos San	Laser therapy for recurrent aphthous stomatitis: an overview	Journal Article	Scopus	
		1		2017	Zand N,Shirkavar	Letter regarding "Effect of laser on pain relief and wound healing of recurrent aphthous stomatitis: a systematic revie	Journal Article	Scopus	
			1	2018	Akerzoul N,Chbic	Low laser therapy as an effective treatment of recurrent aphtous ulcers: A clinical case reporting two locations	Journal Article	Scopus	
	1			2014	André CV,Bosc R	Low Level Laser Therapy in inflammatory and infectious oral diseases [Photothérapie par diode électroluminescente	Journal Article	Scopus	
			1	2013	Anand V,Gulati N	Low level laser therapy in the treatment of aphthous ulcer	Journal Article	Scopus	
1				2016	Babu DB,Chavva	Low level laser therapy to reduce recurrent oral ulcers in behçet's disease	Journal Article	Scopus	
1				2019	Olejnik M,Ślebioc	Low-level laser therapy (LLLT) in the treatment of recurrent aphthous stomatitis (RAS) – A promising treatment optio	Journal Article	Scopus	
1				2020	Ahmed MK,Jafer	Low-level laser therapy and topical medications for treating aphthous ulcers: A systematic review	Journal Article	Scopus	
		1		2020	Slebioda Z,Doroc	Low-level laser therapy in the treatment of recurrent aphthous stomatitis and oral lichen planus: A literature review	Journal Article	Scopus	
		1		2015	Vale FA,Moreira	Low-level laser therapy in the treatment of recurrent aphthous ulcers: A systematic review	Journal Article	Scopus	
		1		2016	Pentapati KC,Sm	Low-level laser therapy versus 5% amlexanox: a comparison of treatment effects in a cohort of minor aphthous ulcer	Journal Article	Scopus	
			1	2016	Spanemberg JC,c	Low-level laser therapy: A review of its applications in the management of oral mucosal disorders	Journal Article	Scopus	
		1		2016	Najeeb S,Khurshi	Management of recurrent aphthous ulcers using low-level lasers: A systematic review	Journal Article	Scopus	
1				2020	Borst J,Ma L	Oral ulcerations in a patient with autosomal dominant hyper-IgE syndrome (AD-HIES)	Journal Article	Scopus	
			1	2019	Kalhori KA,Vahda	Photobiomodulation in Oral Medicine	Journal Article	Scopus	
			1	2016	Pandeshwar P,Ro	Photobiomodulation in oral medicine: a review	Journal Article	Scopus	
				2020	Bardellini E,Vene	Photobiomodulation therapy for the management of recurrent aphthous sto-matitis in children: Clinical effectivenes	Journal Article	Scopus	1
			1	2019	Merigo E,Rocca J	Photobiomodulation Therapy in Oral Medicine: A Guide for the Practitioner with Focus on New Possible Protocols	Journal Article	Scopus	
				2012	Zand N,Fateh M,	Promoting wound healing in minor recurrent aphthous stomatitis by non-thermal, non-ablative CO2 laser therapy: A	Journal Article	Scopus	1
1				2018	Poleník P	Reccurent aphthous stomatis by children [Recidivující aftózní stomatitida u dětí]	Journal Article	Scopus	
1				2015	Ruiz Beguerie J,S	Recurrent aphthous stomatitis	Journal Article	Scopus	
			1	2014	Albrektson M,He	Recurrent aphthous stomatitis and pain management with low-level laser therapy: A randomized controlled trial	Journal Article	Scopus	
		1		2018	Saikaly SK,Saikaly	Recurrent aphthous ulceration: a review of potential causes and novel treatments	Journal Article	Scopus	
		1		2018	Giannetti L,Murr	Recurrent aphtous stomatitis	Journal Article	Scopus	
			1	2009	Zand N,Ataie-Fas	Relieving pain in minor aphthous stomatitis by a single session of non-thermal carbon dioxide laser irradiation	Journal Article	Scopus	
			1	2011	van As G	The diode laser in treating ulcerative oral lesions	Journal Article	Scopus	
				2016	Rezvaninezhad R	The effect Co2 laser on reducing pain associated with aphthous stomatitis	Journal Article	Scopus	1
		1		2020	Nagoro AA,Cecili	The efficiency of ER, CR: YSGG laser and hyaluronic acid gel for recurrent aphthous ulcer treatment	Journal Article	Scopus	1
1				2017	Gerasimova AA,M	Treatment of oral mucosal diseases in workers exposed to harmful factors of production	Journal Article	Scopus	
1				2015	Pavlić V,Vujić-Ale	Treatment of recurrent aphthous stomatitis by laser therapy: A systematic review of the literature [Lečenje recidivira	Journal Article	Scopus	

			1	2017	Yilmaz HG,Albab	Treatment of recurrent aphthous stomatitis with Er,Cr:YSGG laser irradiation: A randomized controlled split mouth c	Journal Article	Scopus	
			1	2022	Ghali HG,Abdulh	Treatment of recurrent minor aphthous stomatitis using diode laser (940 nm)	Journal Article	Scopus	
1				2015	Lal K,Parthiban J,	Usefullness of laser in oral and maxillofacial surgery	Journal Article	Scopus	
1				2015	Babu B,Uppada U	Versatility of diode lasers in low-level laser therapy for the management of recurrent aphthous stomatitis	Journal Article	Scopus	
70	4	289	35	399					16

Literature Review
